# Supplementary material for: Learning potential energy surfaces of hydrogen atom transfer reactions in peptides
Source: Digit Discov. 2026 Mar 30;5(4):1831–44. doi: 10.1039/d6dd00054a (PMC13054793; doi:10.1039/d6dd00054a)
Supplement: DD-005-D6DD00054A-s001 [file DD-005-D6DD00054A-s001.pdf]

Supplementary Information (SI) for Digital Discovery.
This journal is © The Royal Society of Chemistry 2026

SI: Learning Potential Energy Surfaces of Hydrogen Atom Transfer Reactions in Peptides

Marlen Neubert[1,2], Patrick Reiser[1,2], Frauke Gräter[3,4,5,*], and Pascal Friederich[1,2,*]

[1]Institute of Theoretical Informatics, Karlsruhe Institute of Technology, Kaiserstr. 12, 76131 Karlsruhe, Germany
[2]Institute of Nanotechnology, Karlsruhe Institute of Technology, Kaiserstr. 12, 76131 Karlsruhe, Germany
[3]Max Planck Institute for Polymer Research, Mainz 55128, Germany
[4]Heidelberg Institute for Theoretical Studies, Heidelberg 69117, Germany
[5]Interdisciplinary Center for Scientific Computing, Heidelberg University, Heidelberg 69120, Germany
[*]Corresponding author: graeter@mpip-mainz.mpg.de, pascal.friederich@kit.edu

SI 1 Datasets

Table SI 1: Summary of dataset composition used, listing the number of molecular configurations available at the xTB and DFT levels of theory. The datasets are divided into training, evaluation, and test sets for both single molecular systems and linear interpolation tasks.

Dataset type	xTB	DFT
Total	172,042	125,365
Single Systems Training	112,191	65,514
Single Systems Evaluation	7,291	7,291
Single Systems Test	6,836	6,836
Linear Interpolation Evaluation	24,620	24,620
Linear Interpolation Test	21,104	21,104

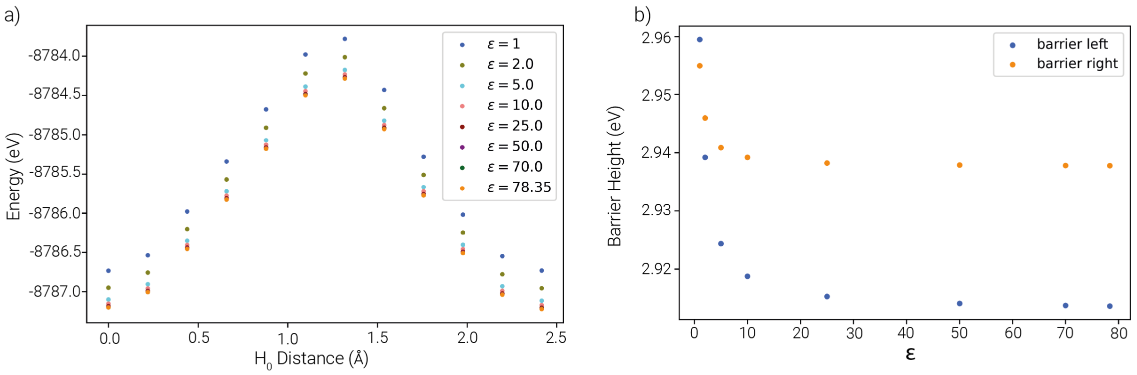

Figure SI 1: Dielectric constant tests for implicit solvent calculations. The dielectric constant (ϵ) used in xTB and DFT calculations was selected based on convergence behaviour of reaction barrier heights and considerations of typical protein environments. a) Example energy profile showing that the barrier height stabilized for $\epsilon > 10.0$. b) Barrier height as a function of ϵ, illustrating convergence beyond $\epsilon = 10.0$.

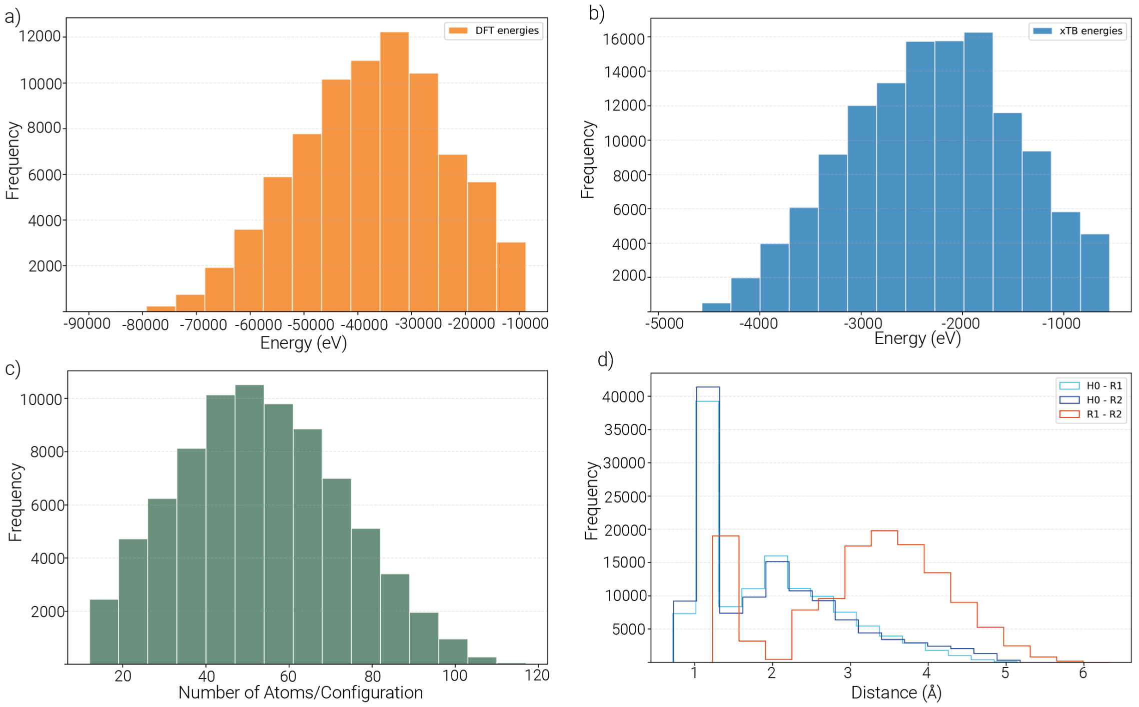

Figure SI 2: DFT and xTB dataset statistics. Potential energy distributions of the configurations of all a) DFT and b) xTB data. Distribution of c) the number of atoms per configuration and d) the Hydrogen atom transferred - radical distances within the DFT dataset.

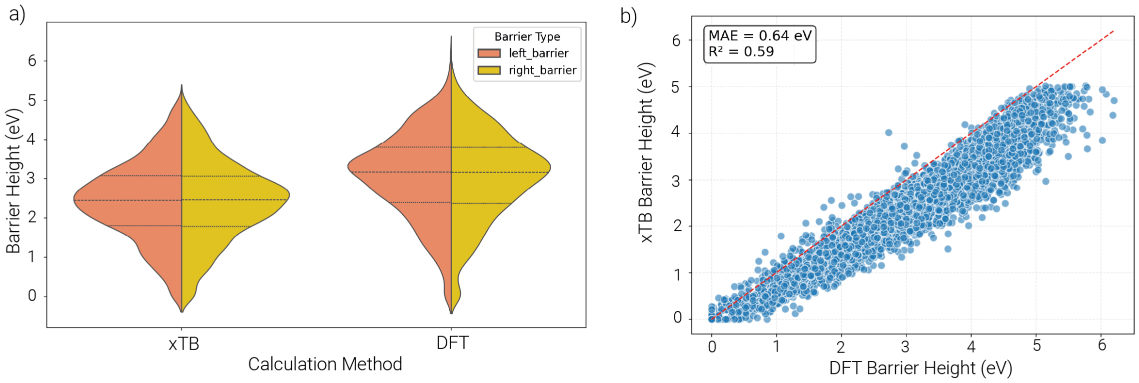

Figure SI 3: Comparison between barrier heights (n = 4,025) calculated using xTB vs. DFT for all configurations of the linear interpolation dataset. a) Violin plot: xTB underestimates both left and right HAT reaction barriers. b) xTB underestimates barrier heights for most systems.

SI 2 Comparative analysis of GNNs

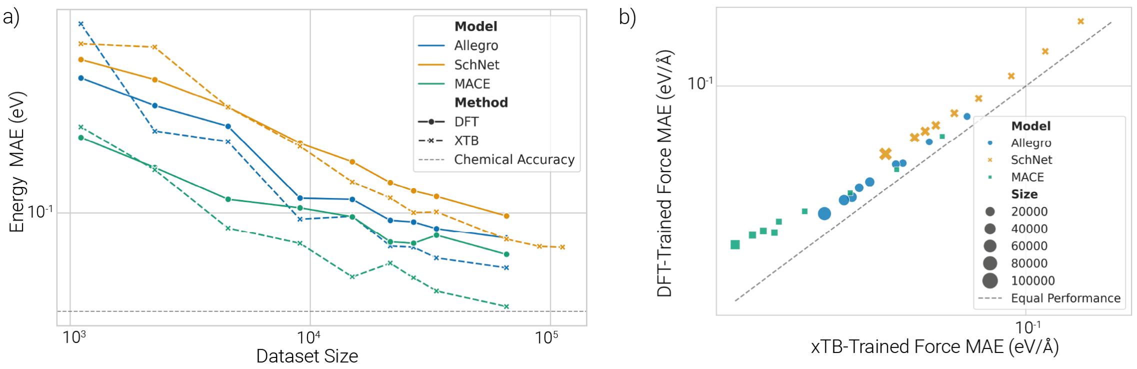

Figure SI 4: Scaling behavior of GNNs: a) Test set energy MAE vs. training dataset size. b) DFT-trained force MAE is higher than xTB-trained force MAE for all trained models.

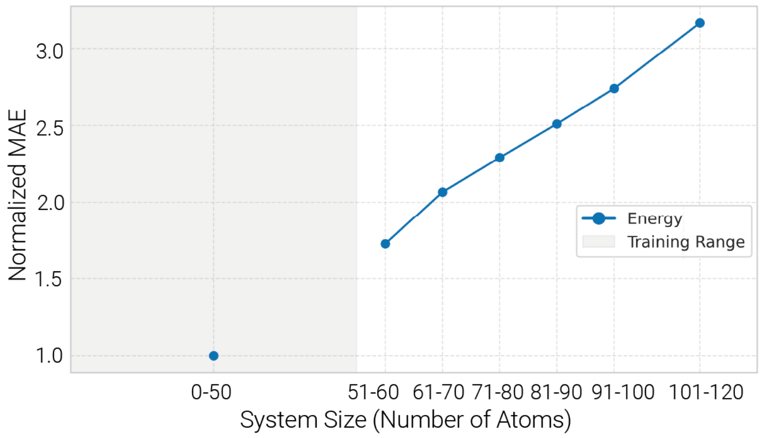

Figure SI 5: Transferability of MACE to different system sizes: Energy MAEs and per-atom energy MAEs vs. atom count. Energy MAE increases with system size, hinting at additive errors.

SI 3 Final model performance

Table SI 2: Test error of models trained on 65,514 xTB configurations and tested on 6,836 unseen configurations and 2,164 barrier evaluations.

Model	Energy MAE (meV)	Force MAE (meV/Å)	Barrier MAE (meV)
SchNet	78	43	78
Allegro	60	30	58
MACE	**42**	**18**	**39**

SI 4 Molecular dynamics simulations

The collagen trajectory used for constructing collagen-derived HAT test systems originates from a collagen model spanning one overlap and one gap region, generated with Colbuilder [1]. The system was simulated with GROMACS 2020 under tensile loading conditions as described in Rennekamp *et al.* 2023 [2]. collagen sequences from *Loxodonta africana*, *Pongo abelii*, and *Rattus norvegicus* were employed, and models included divalent HLKNL and trivalent PYD crosslinks at various positions. Tension was applied by pulling peptide chain ends, using four pulling protocols: (I) pulling from both ends, (II-III) pulling from one side while fixing the other, and (IV) applying forces drawn

from a Gaussian distribution with mean force of $F_{av} = 1nN$ and width $\sigma = F_{av}/3$, while the outer ring of triple helices is pulled at the average force to prevent sliding. From this trajectory, two snapshots at 2 ns and 4 ns were extracted and used as starting structures. Reactive test systems were then generated by selecting local HAT motifs and constructing donor–H–acceptor configurations as described in Sec. 3.5.

We performed molecular dynamics simulations using the final MACE model trained on DFT data within ASE to evaluate the model's dynamical stability and ability to reproduce HAT events. For each of 30 unseen test systems, five replicas were simulated under three conditions: unbiased dynamics, medium steering, and strong steering. Steering was applied along the H-transfer coordinate $q = r(D–H) - r(H–A)$ using a harmonic bias that was linearly ramped and subsequently removed. Simulations employed a Langevin thermostat (NVT) with parameters summarized in Table SI 3. All trajectories were stable and conserved energy within expected thermal fluctuations. A brief transient temperature spike (< 0.5 ps) was observed in two systems; it relaxed quickly and did not affect the subsequent dynamics or outcomes.

Table SI 3: MD settings and outcomes across 30 test systems (five replicas each).

Condition	T (K)	γ (ps^{-1})	Duration (ps)	Bias Q_K	q ramp (Å)	Ramp (steps)	HAT/30	P_{end}
Unbiased	380	0.6	100	–	–	–	6	0.10
Medium steering	410	0.5	50	1.5	$-0.80 \to +0.10$	15000	19	0.51
Strong steering	420	0.5	50	2.0	$-0.90 \to +0.12$	10000	24	0.65

Data availability

The training data, trained models, as well as the code to train all machine learning models and the scripts to reproduce the results of this paper, can be found on https://github.com/aimat-lab/hat_pes_learning (v1.0) and on Zenodo (https://doi.org/10.5281/zenodo.16572631).

References

[1] A. Obarska-Kosinska, B. Rennekamp, A. Ünal and F. Gräter, *Biophysical Journal*, 2021, **120**, 3544–3549.

[2] B. Rennekamp, C. Karfusehr, M. Kurth, A. Ünal, D. Monego, K. Riedmiller, G. Gryn'ova, D. M. Hudson and F. Gräter, *Nature Communications*, 2023, **14**, 2075.
